# Is the positioning of defensive players an aspect to consider in soccer transition games?

**DOI:** 10.5114/biolsport.2026.156229

**Published:** 2026-01-02

**Authors:** Jose A. Asian-Clemente, Bernardo Requena, Luis Suarez-Arrones, Piotr Żmijewski

**Affiliations:** 1 Football Science Institute, FSI Lab, Granada, España;; 2 Department of Sport sciences, Universidad Pablo de Olavide, Sevilla, España;; 3 FC Lugano, Performance Departament, Lugano, Switzerland.; 4 Jozef Pilsudski University of Physical Education in Warsaw, 00-809 Warsaw, Poland

**Keywords:** Football, Tactical, Sprint, High-speed running, Load

## Abstract

This study investigated the influence of defensive player positioning on the external and internal loads experienced during small-sided transition games (TGs) and the subsequent effects on sprint and jump performance. Twenty male youth players (age 15.7 ± 0.2 years; height 175.3 ± 7.5 cm; mass 67.1 ± 6.8 kg) completed two 7-min bouts, with 2 minutes of passive recovery, of 2 vs. 2 TGs under three defensive configurations: defenders in front of attackers (TG_Front_), behind attackers (TG_Behind_), and parallel to attackers (TG_Parallel_). During each TG, we recorded total distance covered (DC), distance covered in running (18.0–20.9 km · h^−1^), high-intensity running (21.0–23.9 km · h^−1^), and sprinting (> 24.0 km · h^−1^), peak speed, mechanical load, and the number of accelerations and decelerations > 1.0 m · s^−2^ and > 2.5 m · s^−2^. Ratings of perceived exertion (RPE) were also collected. Sprint (30 m) and countermovement jump (CMJ) tests were administered immediately before and after each session. The results showed that TG_Parallel_ and TG_Behind_ elicited significantly greater DC, distances in running, high-intensity running, and sprinting zones, mechanical load, accelerations > 2.5 m · s^−2^, and RPE compared to TG_Front_ (p < 0.05). Moreover, TG_Parallel_ produced higher DC in high-intensity running, sprinting, mechanical load, and accelerations > 2.5 m · s^−2^ than TG_Behind_ (p < 0.05). No TG format induced significant changes in sprint or CMJ performance (pre- vs. post-test, p > 0.05). These findings demonstrate that positioning defenders behind or parallel to attackers increases both external and internal loads across running, high-intensity, and sprinting zones during TGs, without compromising subsequent sprint or jump performance.

## INTRODUCTION

Soccer is a sport of a stochastic nature in which players are frequently required to perform in a complex, multi-factorial environment [1, 2]. The nature of the sport suggests that an integrated approach, combining technical, tactical, and physical demands is the most effective way to develop soccer players [3]. This approach is based on the specificity principle, which states that a greater similarity between training demands and sport requirements will result in greater improvements in athletes’ performance [4]. The specificity principle supports the idea that soccer-specific tasks elicit neuromuscular and metabolic adaptations aimed at enhancing players’ physical capacities and fitness, while simultaneously allowing training to be utilized in a more efficient manner [5].

The understanding of training requirements means that currently, soccer training prescriptions follow a game-based approach in which ball drills are focused on the game as a whole [6]. Within this paradigm, the goal is to design drills that simultaneously improve all game components in a specific and integrated context. Traditionally, small-sided games have been considered the most representative soccer-specific task because of their ability to create stimuli similar to a real game [7]. Despite their popularity, these drills can present a possible mismatch with match-play requirements, especially in high-speed and sprint activities if the playing area is not chosen properly [8–10]. An alternative or complementary training method to induce high-speed actions and sprinting exposure consists of running-based drills with linear and non-linear movements [11], but although these strategies are effective for improving physical fitness aspects, they have a limited ability to develop the technical and tactical components. Consequently, designing soccer-specific drills that stimulate high-speed actions remains a key challenge for coaches [12, 13].

Recently, a new type of drill, referred to as transition games (TGs), has emerged in the literature [14–16]. TGs are defined as highly demanding tasks involving running at high speeds, during which players must execute rapid attacks and defend against counter-attack situations, combined with goal shooting [16]. Transitions or counter-attacks are crucial phases of the game, as they have been found to be the most effective style of play for scoring goals, with more goal opportunities occurring during these game phases [17, 18].

In spite of the critical importance of transitions in soccer, they have received limited attention in the scientific literature [15]. Previous research highlighted that TGs induce more high-speed running and higher numbers of accelerations, but with lower distances covered, lower player load and fewer decelerations than small-sided games [14]. As with the small-sided games, these training drills undergo modifications in training load depending on the manipulation of the constraints [15, 16], producing a higher total distance covered, with more high-speed running and longer sprint distances when they are prescribed over larger spaces (larger pitches) and shorter bout durations [15, 16].

Although the amount of research related to TGs has increased in recent years due to their popularity with soccer coaches, there are some issues related to these drills that remain underexplored. Currently, soccer coaches use TGs with different shapes and formats. To train for scoring opportunities in counter-attack actions, they often modify the defensive positioning to enhance players’ abilities in such situations. To the authors’ knowledge, the effects of these modifications on the load and fatigue of the players are unknown.

For this reason, the aim of this study was to examine how variations in the initial positioning of defensive players in TGs influence the external and internal load demands experienced by youth soccer players, and to assess whether these different TG formats elicit acute changes in sprint and jump performance. By systematically comparing three defensive starting positions – front, behind, and parallel – the study aimed to provide practical insights into optimizing the design of TGs to target specific physical outputs within soccer training microcycles.

## MATERIALS AND METHODS

### Participants

Twenty young soccer players (age: 15.7 ± 0.2 years; height: 175.3 ± 7.5 cm; weight: 67.1 ± 6.8 kg) from the professional academy of a Spanish first division club participated in this study. An a priori power analysis (effect size f = 0.6, α = 0.05, power = 0.80) indicated that 18 participants were required; however, 20 were recruited to accommodate practical team-based constraints and ensure sufficient statistical power. Players participated in five training sessions (80–120 min duration) and one competitive match (normally on Sunday) per week. These data were acquired from daily monitoring of workloads during the team’s training, in which player activities are measured over the competitive season [19], so ethics committee clearance was not required. Nevertheless, the study conformed to the recommendations of the Declaration of Helsinki, and the participants were informed of the study’s design and aims, giving their consent before it started.

### Procedures

#### Study design

**FIG. 1:**
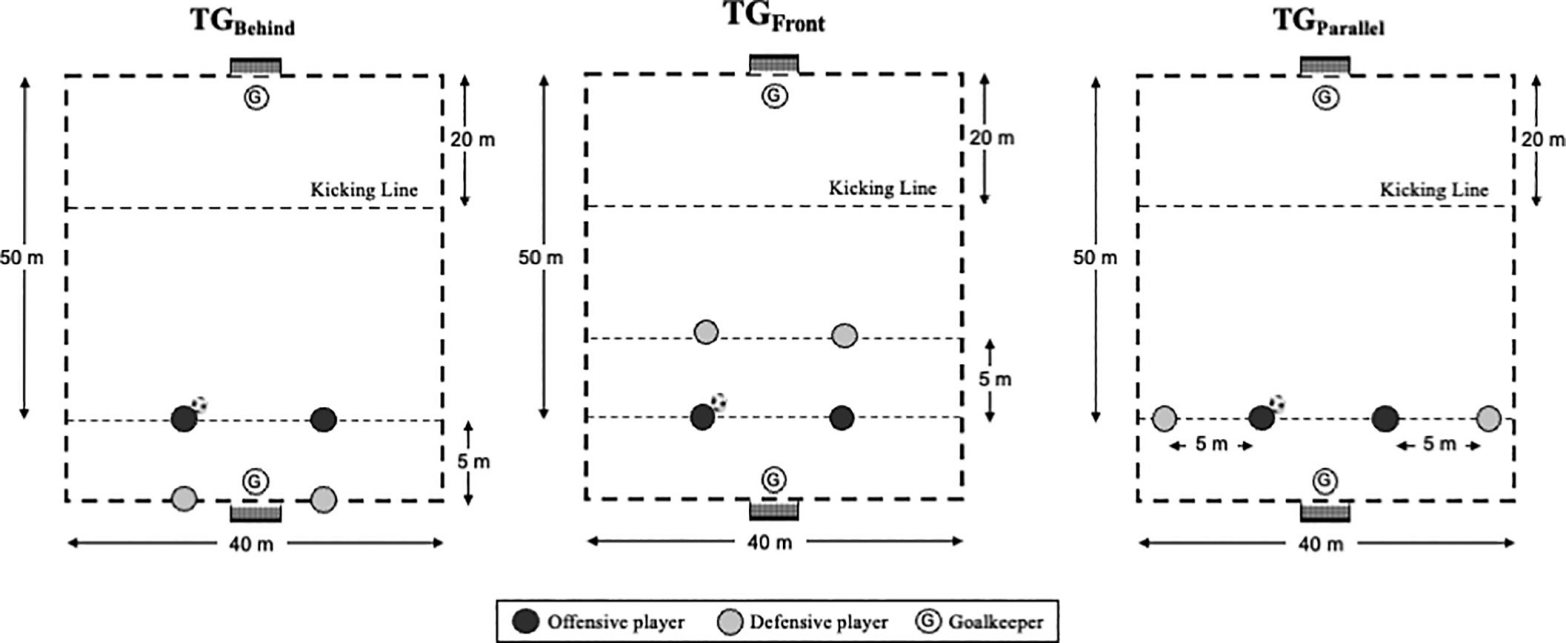
Graphical representation of the transition games (TGs).

The TG designs were selected by two professional coaches holding UEFA Pro Licenses and possessing over 10 years of experience in analysing various match situations, particularly those involving counter-attacks and transitions in soccer. The study intervention lasted six weeks, during which the TGs under study (see Figure 1) were implemented. Across different weeks, players participated in all three 2 vs. 2 TG formats (TG_Front_, TG_Behind_, TG_Parallel_), each being performed twice. To control for potential order effects and ensure balanced exposure, the weekly order of TG formats was pre-determined through randomization prior to the study. In the first three weeks, the formats were presented in the order Week 1: TG_Front_, Week 2: TG_Behind_, and Week 3: TG_Parallel_; during the final three weeks, the order was Week 4: TG_Parallel_, Week 5: TG_Front_, and Week 6: TG_Behind_.

#### Transition games

Following previous studies [14–16], two teams of 10 players engaged in transition games aimed at scoring a goal in a counterattack. Each team was composed of five pairs, and each pair was assigned a role (attacking or defending team). The attacking pairs were tasked with advancing toward the opponents’ goal, while the defending pairs attempted to prevent scoring, starting their movements from the initial positions: A) Front, B) Behind, or C) Parallel. The initial positions of the defenders were chosen based on potential pressures that offensive players may face during matches.

The execution of the three TG formats followed the same procedure: the offensive team attacked the defensive team. After this first attack action – whether the goal was scored or the game ended (the goalkeeper made a save or the ball went out of play) – all the players rested while a new set of teammates began the game. In contrast, if defensive players obtained the ball, they would initiate a quick attack on the opposite goal. Independently of the outcome of this second game action, participants rested, and a new set of teammates would begin play. If in any of the previously described cases, players with possession of the ball did not execute a fast counter-attack, thus converting the play into a positional attack, coaches would finish the game and bring new players onto the field. This dynamic was repeated alternately between teams for the full duration of the TG.

Firstly, teams were assigned to a role (offensive or defensive), and when all their players had participated, the roles were swapped. These TGs consisted of 2 sets of 7 minutes with 2 minutes of passive recovery between sets. If the time ended and some players had not completed the same number of repetitions as their teammates, the drill was continued until all participants had achieved the same number of repetitions. During the three TG formats, the players were divided into balanced teams according to technical and tactical level, competitive experience, player position and the subjective evaluation of the coaches. To minimize variability due to team composition, the same teammates and opponents were maintained throughout the study, with players facing the same opponent pairs. The goalkeepers of the team also participated in the study, but they were not monitored. To avoid any disruption of play, coaches introduced balls as needed and verbally encouraged the players to maintain a high work rate. The soccer players in this study were fully familiar with these games because they had been repeated multiple times during the season, being a frequent drill used by all the academy teams.

#### Jumping and sprinting performance

Before and after the TGs, the jumping and sprint performance of the soccer players were evaluated. The protocol used had previously been applied in a similar study [16]. Before all measurements, participants carried out a 15-minute standardized warm-up consisting of 5 minutes of low-intensity running, 5 minutes of mobility exercises, active stretching, and strength exercises including some jumping drills, and 2 sets of incremental 30-m sprints runs.

Jump ability was evaluated using a vertical countermovement jump (CMJ) with an infrared timing system (Optojump, Microgate, Bolzano, Italy) before and after each soccer drill. A 2-minute recovery period was provided after each transition game prior to the execution of the CMJ. The best of 3 CMJs was chosen for further analysis. Each CMJ attempt was separated by 45 seconds of passive recovery. When 3 valid results were obtained, to avoid a possible fatigue effect, soccer players took a break of 2 minutes before the sprint test. Using photocell gates (Witty, Microgate, Bolzano, Italy), a 30-m standing-start all-out run was tested. Times at the start-finish point and at the 15-m line were included in the analysis. At least 3 valid sprints were performed, separated by 2 minutes of passive recovery. The best trial was recorded for further analysis. During all sprints, verbal encouragement was provided by the coaches to ensure the maximal performance of each player. Two minutes after the speed test, TGs started with the soccer players who had finished the test first. Subjects were familiarized with these tests because they were regularly performed during routine testing at the academy. Four experienced researchers supervised all the interventions to ensure the correct execution of the test. They also collected rating of perceived exertion (RPE) values using a Borg CR10-scale [20]. RPE was recorded on an individual basis before and immediately after completion of each drill. Subjects were educated and familiarized with the use of this scale, which was routinely used by their coaches. This intervention was carried out on Wednesday (Match Day -4 and after a day off) during the initial part of the session. After the intervention, soccer players continued their training session with a ball possession game and a training match.

#### External and internal loads

External and internal loads were monitored using a GPS-based tracking system (Kinexon GNSS, Precision Technologies, Munich, Germany) and RPE. Total distance covered (DC), distance covered between 18.0 and 20.9 km · h^−1^ (DC 18–20.9 km · h^−1^), distance covered between 21.0 and 23.9 km · h^−1^ (DC 21–23.9 km · h^−1^), distance covered at above 24.0 km · h^−1^ (DC > 24 km · h^−1^), peak speed, mechanical load (expressed in arbitrary units), number of accelerations and decelerations above 1.0 m · s^−2^ and 2.5 m · s^−2^ (Acc > 1.0 m · s^−2^, Acc > 2.5 m · s^−2^, Dec > 1.0 m · s^−2^ and Dec > 2.5 m · s^−2^), and RPE were recorded to quantify the external and internal loads. These variables have been used previously in the literature [14–16].

### Statistical analysis

Data are presented as mean ± standard deviation (SD). All variables showed a normal distribution (Shapiro-Wilk Test). To evaluate the effects of the different TG formats on internal and external load variables, a repeated-measures analysis of variance (ANOVA) was conducted, including the analysis of interaction effects between TG formats and time points (pre vs. post) where applicable. When significant main or interaction effects were detected, Bonferroni-adjusted post hoc tests were applied to identify pairwise differences. Differences in sprint and jump performance before and after each TG were analysed using Student’s paired-sample t-tests. Statistical significance was set at p ≤ 0.05. All statistical analyses were performed using SPSS Statistics (version 19, IBM Corp., Armonk, N.Y., USA). Cohen’s effect sizes (ES) with 95% confidence intervals (CI) were also calculated to quantify the magnitude of differences, with thresholds defined as follows: trivial (< 0.20), small (0.20–0.59), moderate (0.60–1.19), large (1.20–1.99), and very large (≥ 2.0), as recommended by Hopkins et al. [21].

## RESULTS

**FIG. 2:**
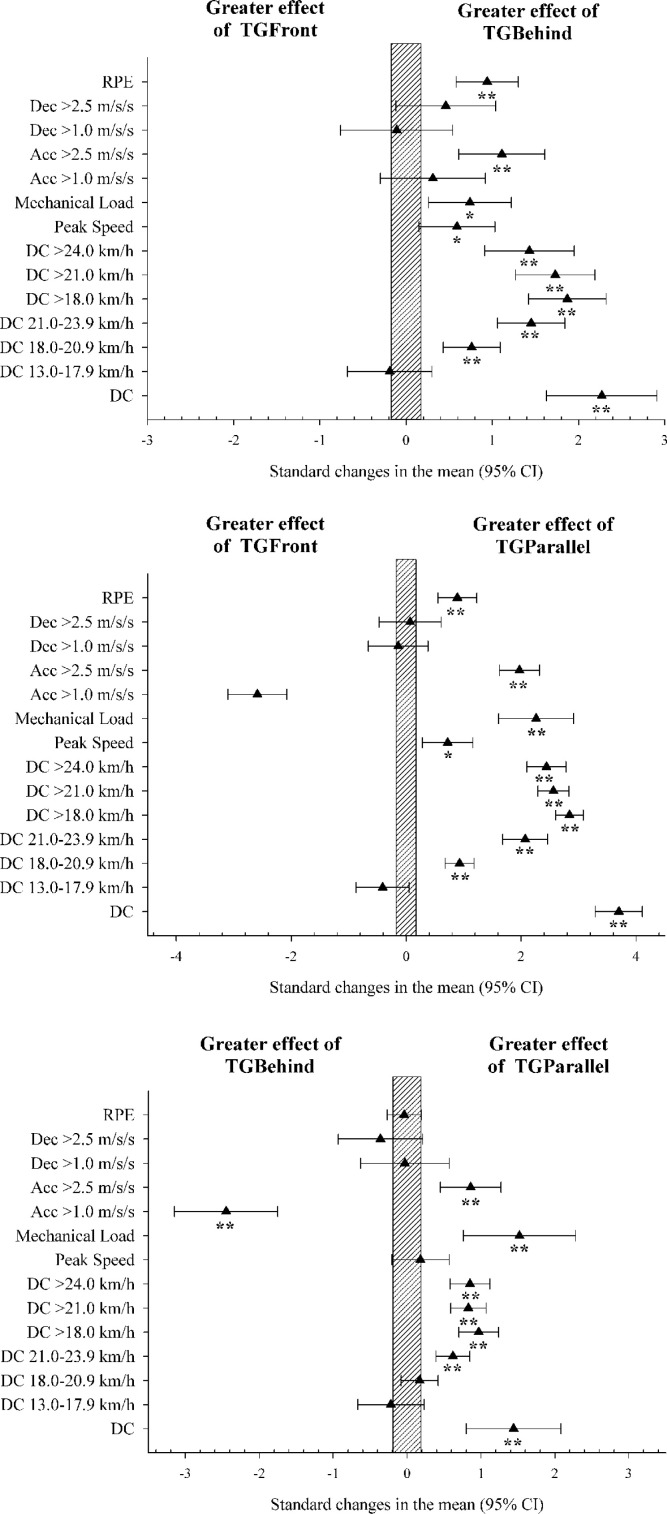
Effect size comparison between transition games. TG = transition games; DC = distance covered; Acc = accelerations; Dec = decelerations; RPE = rating of perceived exertion. * *p* < 0.05; ** = *p* < 0.01.

Table 1 and Figure 2 show the internal and external load data from each TG analysed. TG_Parallel_ showed significantly higher DC, DC 21.0–23.9 km · h^−1^, DC > 24 km · h^−1^, mechanical load and Acc > 2.5 m · s^−2^ than TG_Front_ and TG_Behind_ (*p* < 0.01). Moreover, TG_Parallel_ reached significantly higher DC 18.0–20.9 km · h^−1^ (*p* < 0.01), peak speed (*p* = 0.01) and RPE (*p* < 0.01) than TG_Front_ (*p* < 0.01), but lower Acc > 1.0 m · s^−2^ compared with the other drills examined (*p* < 0.01). Likewise, TG_Behind_ showed significantly greater DC, DC 18.0–20.9 km · h^−1^, DC 21.0–23.9 km · h^−1^, DC > 24 km · h^−1^, peak speed, mechanical load, Acc > 2.5 m · s^−2^ and RPE than TG_Front_ (*p* < 0.01 in all variables except peak speed, *p* = 0.03). The variables DC 13.0–17.9 km · h^−1^ and Dec > 2.5 m · s^−2^ did not show statistically significant differences (*p* > 0.05).

**TABLE 1:** Comparison of external and internal loads of transition games analysed.

	Mean ± SD			TG_Front_ vs TG_Behind_	TG_Front_ vs TG_Parallel_	TG_Behind_ vs TG_Parallel_

Variable	TG_Front_	TG_Behind_	TG_Parallel_	*p*	*p*	*p*
DC	1511.2 ± 133.6	1881.4 ± 260.0	2136.6 ± 184.8	< 0.01	< 0.01	< 0.01
DC 13.0–17.9 km · h^−1^	217.9 ± 49.8	212.2 ± 60.1	198.5 ± 50.6	0.52	0.14	0.40
DC 18.0–20.9 km · h^−1^	97.5 ± 24.5	119.4 ± 30.4	120.8 ± 24.3	< 0.01	< 0.01	0.27
DC 21.0–23.9 km · h^−1^	66.2 ± 26.0	124.9 ± 24.4	164.4 ± 41.6	< 0.01	< 0.01	< 0.01
DC > 24.0 km · h^−1^	44.2 ± 34.1	168.1 ± 83.2	305.5 ± 78.5	< 0.01	< 0.01	< 0.01
Peak speed	28.4 ± 2.0	29.6 ± 1.5	30.0 ± 1.3	0.03	0.01	0.43
Mechanical load	386.6 ± 45.8	423.2 ± 50.0	507.3 ± 128.7	0.01	< 0.01	< 0.01
Acc > 1.0 m · s^−2^	7.4 ± 3.3	6.8 ± 5.3	2.0 ± 1.3	0.39	< 0.01	< 0.01
Acc > 2.5 m · s^−2^	8.8 ± 3.0	14.5 ± 5.7	19.4 ± 3.1	< 0.01	< 0.01	< 0.01
Dec > 1.0 m · s^−2^	6.4 ± 2.2	6.6 ± 3.5	6.2 ± 2.6	0.78	0.65	0.85
Dec > 2.5 m · s^−2^	6.4 ± 3.1	8.2 ± 3.7	7.4 ± 3.3	0.18	0.83	0.29
RPE	7.0 ± 0.7	7.7 ± 0.6	7.6 ± 0.5	< 0.01	< 0.01	0.74

SD = standard deviation; TG = transition games; *p* = *p* value; DC = distance covered; Acc = accelerations; Dec = decelerations; RPE = rating of perceived exertion.

Data for the tests performed and the interaction effects between groups are shown in Table 2. For 15-m and 30-m sprinting as well as for CMJ, no statistically significant main time effects or interactions were found (all *p* > 0.1)

**TABLE 2:** Sprinting and jumping performance before and after selected transition games.

	TG_Front_		TG_Behind_		TG_Parallel_		Time	Time × group interaction

	Pre	Post	Pre	Post	Pre	Post		
Sprint 15-m (s)	2.44 ± 0.10	2.50 ± 0.10	2.42 ± 0.14	2.43 ± 0.12	2.45 ± 0.09	2.43 ± 0.10	0.413	0.160
Sprint 30-m (s)	4.28 ± 0.15	4.34 ± 0.15	4.19 ± 0.11	4.25 ± 0.19	4.27 ± 0.14	4.28 ± 0.14	0.067	0.537
Jump test (cm)	37.51 ± 3.90	37.95 ± 5.01	37.50 ± 4.39	36.82 ± 5.08	37.21 ± 4.79	36.60 ± 4.94	0.684	0.789

Values (mean ± SD) SD = standard deviation; TG = transition games.

## DISCUSSION

This study is the first to experimentally isolate the effect of defender positioning in TGs on both performance load and neuromuscular recovery, offering practical insights into training task design for load manipulation in youth soccer. The primary finding was that when defenders were positioned behind or parallel to the attackers during a TG, the exercise elicited greater DC, DC 18.0–20.9 km · h^−1^, DC 21.0–23.9 km · h^−1^, DC > 24 km · h^−1^, peak speed, mechanical load, Acc > 2.5 m · s^−2^ and RPE, compared to TGs in which the defenders were positioned in front of the attackers. Secondly, this study showed that no significant interaction effect was observed between TG format and time (pre-post) for sprinting or jumping performance, suggesting that all formats induced similar fatigue profiles despite differences in external load.

This is the first study to analyse the positional influence of the defenders in a counter-attack exercise such as a TG. The results showed that defensive positioning is a key element in TG design, since the initial starting point determines statistically significant differences in both the external and internal training loads. Beginning the TG exercise with players facing each other (TG_Front_) decreased the external and internal load compared to defensive starting positions either behind (TG_Behind_) or parallel (TG_Parallel_). This may be explained by the defensive players during each of the TGs. Defending in a block between the attacker and the goal and reducing the pressure area are effective strategies for increasing defensive success in soccer [22, 23]. Such strategies could have led defenders in the TG_Front_ formation to adopt more conservative behaviour, focusing on protecting the space around the goal, thereby simulating what occurs in other specific tasks such as small-sided games [24]. To achieve a similar advantage, defenders in the TG_Behind_ and TG_Parallel_ formations had to exert greater effort. Because their starting position was not advantageous, they displayed higher external load during these drills. Likewise, offensive players may have taken advantage of the absence of defenders in their direct path, intensifying their efforts to capitalize on shooting opportunities presented by the goalkeeper’s unique positioning. TG_Parallel_ was also more demanding than TG_Behind_. This could be because, as previously argued, defenders starting in parallel encountered no opposition in their path, whereas defenders starting from a delayed position found the offensive player in their trajectory, potentially limiting available space to run and reducing the intensity of effort. In this latter case, the offensive players could have also used their advanced position to protect the ball with their body, slowing down the task compared to TG_Parallel_. A previous study demonstrated that during TGs, a greater available space promoted a higher total and high-speed distance covered [15]. The same study also showed that smaller available space was associated with greater Acc > 1.0 m · s^−2^ [15], which could explain the higher numbers of low-intensity accelerations observed during TG_Behind_ and TG_Front_ compared to TG_Parallel_.

Our results are in line with previously published articles about TGs, confirming that manipulating the constraints in these drills affects their external and internal loads [15, 16]. TGs performed with shorter bout durations and larger depths were effective for training high-speed running and sprinting [15, 16]; therefore, coaches could include TG_Behind_ and TG_Parallel_ in this proposal, since they impose substantial high-speed running demands. For example, DC > 24 km · h^−1^ in these TGs ranged from 168 to 305 m, while previously the highest mean values reported were 121–156 m in the most intense studied tasks [14, 15]. Likewise, peak speed values reached 29.6–30.0 km · h^−1^, while in previous studies the peak speed was 28.8 km · h^−1^ [14, 15]. The shorter duration of the repetitions during TG_Behind_ and TG_Parallel_ (< 10 s) may result in lower muscle fatigue and metabolic stress [16], allowing players to produce more intense efforts.

By contrast, the present study produced different results compared with an earlier investigation that analysed the times for the 15-m and 30-m sprints and the CMJ heights after TG. While previously it was reported that the running time and jump performance of soccer players were reduced after 15, 30 and 60 s of TGs [16], our results did not reveal significant effects of TG_Front_, TG_Behind_ and TG_Parallel_ on sprint or jump performance (*p* = 0.07). This observation should be interpreted with caution given the lack of statistical significance. Differences between studies might be partly related to the greater perceived fatigue reported by players in the previous investigation compared to the present one (9 vs. 7 RPE values, respectively). Although speculative, shorter execution times in the current drills may have limited the depletion of high-energy phosphates and alterations in muscle pH [25–27], thereby reducing fatigue. However, since no biochemical or electromyographical data were collected, these interpretations remain hypothetical and should be confirmed in future research.

Offensive transitions have a profound impact on goal-scoring patterns in modern football. Analysis of top-tier leagues reveals that a significant proportion of goals, often exceeding 53%, originate from such phases of play [28]. This underscores the paramount importance that training this phase of the game should have in modern football. Although the current study provides novel insights into the factors determining external load during transition drills, offering a practical framework for optimizing training prescription, future research should build upon these findings by considering additional variables that may help coaches gain a deeper understanding of this type of task. For example, given that the goalkeeper plays an increasingly important role in teams’ offensive organization in modern football [29], it would be relevant to examine how the goalkeeper’s involvement influences the dynamics and external load of TGs.

### Limitations

Although the results of this research appear to contribute to the knowledge of TGs, some limitations should be considered when interpreting the data. The TGs were developed by analysing the acute effects of these tasks in a group of young professional soccer players; therefore, caution should be exercised when extrapolating these findings to longterm contexts or to other populations. For this reason, future studies should be conducted using a longitudinal design to examine long-term adaptations in young professional soccer players or in other populations, such as professional female soccer players or adult male professionals. Likewise, it should also be emphasized that, during the statistical analysis, data were considered as a whole, without distinguishing the load associated with the players’ participation in each role (offensive and defensive). Consequently, it remains unclear whether defenders and attackers experience similar demands during TGs or if one specific phase imposes greater requirements. Therefore, future studies should address this issue by analysing training loads during TGs while differentiating between offensive and defensive roles. Similarly, it should be noted that in this study the duration of each repetition was not quantified according to the type of transition game. Since it remains unclear whether these transition games varied in duration across executions and whether such variation could impose different demands on soccer players, future studies should monitor this aspect. Finally, it is important to acknowledge that the psychological response, tactical decision-making load, and objective indicators of internal load (e.g., heart rate) associated with these tasks were not analysed. Since these factors may influence the ecological validity of the findings, future research should consider incorporating them when designing and evaluating transition-based tasks.

### Practical applications

This study offers soccer coaches objective evidence on how defender positioning can be used as a novel constraint in TGs. Positioning defenders behind (TG_Behind_) or parallel (TG_Parallel_) to attackers during the microcycle’s acquisition phase (i.e., 3–4 days before a match) effectively increases high-speed running, sprint distances and high-intensity accelerations. In contrast, placing defenders in front (TG_Front_) reduces the overall physical load, making it ideal for practising counter-attack scenarios or for tapering sessions 1–2 days prior to competition. Finally, since each TG format involves only a small number of players, all three (TG_Front_, TG_Behind_ and TG_Parallel_) can be easily incorporated into post-match compensatory sessions for non-starters or players with limited playing time.

## CONCLUSIONS

The findings of this study demonstrate that defender positioning during TGs significantly alters both the external and internal loads experienced by soccer players. When defenders are placed behind or parallel to the attackers, players achieve greater DC, DC at 18.0–20.9 km · h^−1^, 21.0–23.9 km · h^−1^, and > 24 km · h^−1^, as well as increased peak speed, mechanical load, and Acc > 2.5 m · s^−2^, accompanied by elevated RPE, compared with when defenders are positioned in front of the attackers. Conversely, none of the TG formats significantly affected subsequent sprint or CMJ performance under the tested conditions.
